# American Hospitals for the Insane
*Reports of Institutions for the Insane in the United States.1. Of the Pennsylvania Hospital for the Insane, for the years 1851 and 1852.2. Of the Massachusetts State Lunatic Hospital, for the years 1851 and 1852.3. Of the Bloomingale Asylum, for the years 1851 and 1852.4. Of the Ohio Lunatic Asylum, for the year 1851.5. Second and Third Biennial Reports of the Illinois State Hospital, for the years from 1849 to 1852.


**Published:** 1853-10-01

**Authors:** 


### AMERICAN HOSPITALS FOR THE INSANE.*

NOTICES of most of the Annual Reports coming from the establishments devoted to the treatment of the insane in this country, down to the close of the year 1850, have already appeared in our columns. Our file of these documents is not complete to the present time, the superintendents of some of the hospitals having failed to forward them to us. We proceed, however, to review such of

* Reports of Institutions for the Insane in the United States.

1. Of the Pennsylvania Hospital for the Insane, for the years 1851 and 1852.
2. Of the Massachusetts State Lunatic Hospital, for the years 1851 and 1852.
3. Of the Bloomingdale Asylum, for the years 1851 and 1852.
4. Of the Ohio Lunatic Asylum, for the year 1851.
5. Second and Third Biennial Reports of the Illinois State Hospital, for the years from 1849 to 1852.

them as we are enabled to do without overleaping a year, and thus making a hiatus in their history, as represented on our pages.

1. *The Pennsylvania Hospital for the Insane.*—According to the report for 1851, the number of patients admitted into this Institution in the course of

| | |
|---|---|
| the year, was | 204 |
| Remaining at the close of 1850 | 213 |
| Whole number for the year | 417 |
| Discharged or died | 201 |
| Remaining at the close of 1851 | 216 |
| Of the patients discharged, there were cured | 107 |
| Died | 26 |

*Causes of death.*—Acute mania 8, acute dementia 1, softening of the brain 5, epilepsy 1, pulmonary consumption 1, dysentery 3, chronic ulceration of throat 1, exhaustion from long refusal of food 1, suicide 1, cancer 1, old age 2.

"Several of the cases of acute mania were undoubtedly injured by the journey to the hospital during the existence of the acute symptoms, which often cannot without difficulty be distinguished from those of inflammation of the brain. While this doubt exists, the patient had better be retained at home, and the probabilities of ultimate recovery are not lessened by such a course.

"The premature removals have this year been rather less frequent than heretofore, and there is reason to believe that the importance of persevering in a course of treatment for insanity is beginning to be more generally understood."

The principal improvements in the establishment during the year, are a new museum and reading-room, additional means to extinguish fire, in case of conflagration, a summer-house in the women's pleasure-grounds, and the substitution of steam for horse-power in supplying water. The new museum is constructed upon the same model as the one previously erected, and the two form a symmetrical feature in the arrangement of the buildings.

"The whole arrangement of the new one is such as to make it an attractive place of resort, especially to the convalescent, and the cultivated, studious patient; to all, indeed, who desire a cheerful and comfortable apartment, where they can quietly read and study, or amuse themselves by inspecting the various objects of interest spread before them. The funds which have been contributed have not only enabled us to put up and finish the building, but also to procure a very fair beginning for a library, and a foundation for a collection of specimens of natural history, minerals, shells, &c., and some pictures and busts. A fine dioptric prismatic lantern and a microscope have also been procured from the same source."

If there be any dwelling inhabited by human beings in which a conflagration is more fearful than in any other, it is, and for very obvious reasons, an establishment for the insane. This evident truth, together with the fact that one of our public asylums was burned but two or three years since, and within the last three months a private one, while on the Continent of Europe several have been destroyed in a similar manner, should induce all who have the charge of such institutions to see that the means of protecting them from fire are ample and efficient. Hence, the following remarks of Dr. Kirkbride may not be inappropriate in this place.

"Ordinarily, the greatest danger to be apprehended from fire in such establishments, is not so much that the inmates may be burned as from suffocation; and of course their safety consists especially in well-devised plans for prevention, or, if that cannot be, of prompt detection, with abundant means always in order, for immediately extinguishing it. To effect these objects properly, the subject should be prominent in the minds of those who originally control the character of the edifice, quite as much as of those who are subsequently to manage it. It would seem to require little argument to prove that all such buildings should be made as nearly fire-proof as circumstances will permit. If it is not deemed advisable to arch them throughout, other expedients should be adopted to prevent the rapid spread of fire, and to expedite the escape of the inmates. All the stairways should be made of iron or other indestructible material, ample in size and number: the roof should be of metal or slate, and arrangements should be made at different points, by which, if a fire does occur, it can be confined to one section of the building. There should also be a mode provided, by which, if at such a time smoke should enter the air-chambers below, it could be prevented from rising through the flues in a dangerous amount to the wards above. All such establishments should be warmed by fresh air passed over steam or hot-water pipes in air-chambers in the cellar, with the boilers placed in a building entirely detached from the main structure, and some distance from it. This mode of heating, carried out in the way suggested, will, of itself, remove the greatest source of accidents from fire in public institutions."

In the Report for 1852, it is said, that "Notwithstanding the extensive provision for the insane made by the State, at Harrisburg, and which has been available during the year just closed, this institution has been about full during the whole period, and for much of the time inconveniently crowded, particularly in the wards appropriated to men."

| | |
|---|---|
| Patients remaining at the close of 1851 | 216 |
| Admitted in the course of the year | 197 |
| Whole number | 413 |
| Discharged or died | 198 |
| Remaining at the close of 1852 | 215 |
| Of the patients discharged, there were cured | 99 |
| Died | 28 |

*Causes of death.*—Acute mania 5, pulmonary consumption 6, softening of the brain 6, congestion of the brain 3, exhaustion from long continued refusal of food 2, sloughing of the perineum 1, suicide 1, convulsions 1, inflammation of intestines 1, chronic ulceration of intestines 1, foreign body entering the trachea 1.

"Five of the cases terminated within ten days of their admission, and these were the fatal cases of acute mania."

Seven of the cases discharged were removed before they had been subjected to treatment sufficiently long to test their curability.

The whole number of cases received since the opening of the institution, is, of males 1212, females 995—total, 2207. Of these, 1049 have been discharged cured, and 230 died.

As the subject is of much importance, no apology is necessary for introducing a considerable portion of the very judicious and just remarks upon bodily restraint, which are found in the report before us.

"No point connected with the treatment of the insane is now more conclusively established than that every such institution *may* be conducted without the use of any mechanical restraint whatever. Whether it is expedient to do so, under all circumstances, is not so well settled. To dispense with restraining apparatus entirely, requires that a hospital should be so constructed as to give all the benefits of the most perfect classification, that it should always have a force of intelligent trained attendants, and abundant means for exercise and occupation in the open air. . . . . It is no advance to give up restraining apparatus, and substitute frequent and long-continued seclusion. An individual may really be more comfortable and much better off in the open air, with some mild kind of restraining apparatus on his person, than he would be confined to his chamber without it. . . . . . Temporary seclusion to a chamber, however, is a remedy not to be dispensed with, and is really important; but those who control it, should especially endeavour to make the periods of its use as short as possible, and always remember that from the moment it ceases to be useful, it rarely fails to become injurious. The free use of restraining apparatus is unfortunate in its direct effects upon the patients, for it brings about bad habits, and prevents the use of valuable means of treatment. It is, perhaps, still more mischievous, by its bad effects upon the attendants, and all those who have the care and control of the inmates of an hospital. Where restraining apparatus is kept in the wards, and those in them become accustomed to seeing and using it, it soon comes to be regarded as the great resource in times of difficulty and danger, and is liable to make us forget the great importance of what can only be called tact, and the happy influence of gentleness, kindness, and sympathy, which, with occupation, constitute the great moral remedies for all forms of this affection.

"Desirable, or at least convenient, as physical strength is, in an hospital for the insane, no one can be long in such an institution without discovering that those who exercise most control over patients, exert the most powerful restraining influences, and are most reliable with the excited, and most judicious in the time of difficulty, are not the individuals who depend upon their strength, whatever it may be, but are often the very persons who physically could render but little service. The gentleness and quiet confidence of a child may, under certain circumstances, effect what the strong man might have to give up in despair.

"Regarding a large and varied supply of restraining apparatus as an undesirable possession for any hospital, and believing the devising of new forms of it an unfortunate use of that ingenuity which should be employed in contriving means for dispensing with it entirely, this hospital has never owned a strait-jacket, a muff, a tranquillizing chair, or any of the still harsher means formerly used, nor of the novel ones more recently recommended. . . . . . . For the whole period of its existence, the average number using restraining apparatus would not exceed one per cent. of those in the house, and not more than from four to six in temporary seclusion in their chambers, generally for periods of from a few hours to a fraction of an hour. It has frequently happened that for several months together there has been no restraining apparatus used in the institution. When apparatus is used, it is either in the form of leather wristbands, secured by a belt around the body, soft leather mittens fastened in the same way, a strong dress with the sleeves connected, or the apparatus for confining a patient on his bed."

After remarking that such restraints are sometimes evidently useful, that in certain forms of insanity they may even save the life of the patient, and that the sole direction of the apparatus should lie with the physician, Dr. Kirkbride says,—"It may not be amiss to refer to the great advantage which is experienced in hospitals for the insane, from doing away, to the utmost possible extent, with even the *appearance* of restraint. This is the true field for the ingenious, and their efforts and contrivances in this way can hardly do harm. It begins in the very choice of a site; it continues in the construction of the buildings, in the arrangement of the wards, in their furniture and fixtures, in the kind and position of the inclosures, and in the conveniences, comforts, and luxuries of the establishment. It consists in making other objects prominent, even where restrictions are intended; in masking, as far as may be, the unpleasant part of what is unavoidable, and in bringing out in bold relief before the patients so many objects for agreeable contemplation, and so many modes of occupation, as to leave little time or inclination for dwelling on what is of a less pleasant character."

A "Calisthenium"—a novelty, for females, in establishments for the insane, and therefore worthy of being heralded—containing a bowling-alley, and other means of exercise, has been erected for the women's department of the institution. It is 60 feet long by 9 in width. In connexion with the description of this, Dr. Kirkbride makes the following remarks:—

"There is a large class of nervous affections, from the slightest shades of diseased health up to diseases of the gravest character, which are mainly owing to a continued violation of natural laws, few of which can long be trampled on with impunity. Prominent among these laws seem to be those provisions which require that man should make free use of his muscles, and have pure air for the purposes of respiration. . . . . It is unquestionably the great misfortune of many studious men and women, and of others with different sedentary occupations, that their pursuits present almost insuperable obstacles to their using free exercise in the open air, although it may well be doubted whether a few hours thus spent in every day would not, at the end of the year, have enabled them, with less waste of the vital energies, to have accomplished an equal amount of work, and, at the same time, to have laid up a capital of health for future emergencies. . . . . It is desirable that every part of the human body should be harmoniously cultivated; but that which will most tend to keep down an unduly excited nervous system, will unquestionably be found to be a proper development and exercise of the muscular. 'Muscles *versus* Nerves,' is really the motto of our new Calisthenium, and the Calisthenium has been established from a conviction that a large number of cases of the highest interest are constantly to be met with, attributable to the causes already referred to, and proving conclusively the truth of the views which have just been cursorily given."

Gas, from the Philadelphia gas-works, has been introduced into the building, which is furnished with two hundred and forty-five burners. "In the lecture-room, especial pains have been taken to make it tributary to the objects for which that room is designed—the amusement and instruction of the patients. Lights have been arranged to show transparencies to great advantage, while fixtures have been contrived for the convenient darkening of the room during the exhibition of dissolving views, and for showing the different modes in which gas may be burned, as well as some other somewhat novel arrangements that are likely to interest our audience."

2. *Massachusetts State Lunatic Hospital.*—In Massachusetts, a new State Hospital for the insane is nearly completed. Dr. Chandler, of the hospital at Worcester, in his report for 1851, in alluding to it, and also to the prospect that a third establishment of the kind will become necessary in the course of a few years, discusses the subject of having distinct institutions for the two sexes. He quotes the great German *psychiater*, Dr. Jacobi, in favour of such a plan, and concludes that the propriety of it will hardly be questioned by those who are practically familiar with the subject.

| | Men. | Women. | Total. |
|---|---|---|---|
| Patients at the State Hospital, Dec. 1, 1850 | 228 | 213 | 441 |
| Admitted in the course of the year . . | 125 | 138 | 263 |
| Whole number . . . . . . . . | 353 | 351 | 704 |
| Discharged and died . . . . . . | 111 | 127 | 238 |
| Remaining, Nov. 30, 1851 . . . . . | 242 | 224 | 466 |
| Of those discharged, there were cured . . | 56 | 55 | 111 |
| Died . . . . . . . . . . . | 13 | 26 | 39 |

*Causes of death.*—Marasmus 3, apoplexy and palsy 6, consumption 5, epilepsy 4, suicide 1, disease of brain 1, typhus fever 1, lung fever 2, dysenteric fever 1, inflammation of bowels 3, erysipelas 3, bronchitis 1, old age 1, maniacal exhaustion 5, pleurisy 1, jaundice 1.

"Intemperance," says Dr. Chandler, "sends a few of its victims to us every year. A singular case, in respect to long-continuance, now nearly a year, of the peculiar symptoms of delirium tremens, has been under our care. When his attention is not diverted by the presence of others, he almost constantly sees 'the pistols of the villains who are trying to shoot' him, 'pointing right towards'

him. He often holds the building from 'tipping over on to him,' as he says; and he will exert himself for hours to prevent his wagon from turning over."

"Two cases have come to us the past year, resulting from disease of the bowels contracted in California. The disease of the bowels in each case has been removed, but the sympathetic affection of the brain will remain. Within the last three years, the wives of nine men have come to our care in consequence, in almost every instance, of their husbands going to California."

In the remarks upon the mortality in the institution, it is stated that "insanity, with many, is but one of the symptoms of a general breaking-up of the physical constitution." It "usually consumes the vital principle rapidly. But there are a few exceptions where the physical powers are but little affected by many years of mental disturbance. The average of the 36 who died the last year, the duration of whose insanity was known, was 49 years and 8 months. The average time insanity continued in the 28 in whom it lasted more than one year, was 9 years and 3 months; and the average time it continued in the 8 in whom it did not continue one year, was 4 months and 7 days."

A valuable meteorological journal has been published in the annual reports of this hospital for the last thirteen years. In 1851, the State furnished the institution with a set of instruments for the purpose of making more extensive observations. They consist of a barometer, thermometer, psychrometer, rain-gauge, and graduated measures. They were accompanied by a table of reduction, and the directions emanating from the Smithsonian Institution.

By the report of 1852, it appears that this establishment is likely to become an asylum for foreign paupers, rather than a curative establishment for natives. The number of foreigners remaining at the close of the year 1842 was 34, and the number has regularly increased, until, at the end of the last year, it was 241.

| | Men. | Women. | Total. |
|---|---|---|---|
| Patients remaining, Dec. 21, 1851 | 242 | 224 | 466 |
| Admitted in the course of the year | 148 | 161 | 309 |
| Whole number | 390 | 385 | 775 |
| Discharged | 126 | 117 | 243 |
| Remaining, Nov. 30, 1852 | 264 | 268 | 522 |
| Of those discharged, there were cured | 55 | 48 | 103 |
| Died | 20 | 25 | 45 |

*Causes of death.*—Maniacal exhaustion 11, marasmus 7, consumption 6, apoplexy and palsy 3, epilepsy 3, "disease of brain" 2, old age 6, disease of heart, suicide, hemorrhage, intestinal inflammation, dropsy, diarrhœa, and 'disease of brain from intemperance," 1 each.

During the first half of the year, there were 13 cases of erysipelas of the head among the patients, but none of them were fatal. The application of warm water was "grateful in some cases, but the effects of other topical applications, of doubtful utility."

In reference to the subject of hereditary predisposition to mental alienation, Dr. Chandler says: "A mother, two sisters, and a brother, have been inmates of this hospital. All of them have been here twice, and some three times. Two brothers and two sisters, of another family, have been inmates here. Other members of the same family have been insane. The members of this family were periodically affected. Of another family, two brothers and a sister have been patients here. The father, mother, a sister, and two other brothers, were for a time insane. At this time we have three male patients, who have each a sister with us. A mother and her daughter are patients here, at this time. We once had twin sisters, and once a man and his wife, as patients."

Upon another subject, and one which has attracted considerable attention in some other parts of the country, during the past two or three years, we find the following statements: "Five (patients) have been brought to us, whose minds were overborne by investigating the phenomena early designated as the 'Rochester knockings.' The two males recovered their former health of body and mind very favourably, and two of the females will probably recover. They were all what are called 'nervous' persons, and were, at the time of their admission, suffering from physical disease."

The whole number of patients admitted during the twenty years of the operations of the hospital, is, of males 2090, females 2080, total 4170. Of these, 929 males and 979 females, have been discharged recovered; and 232 males and 218 females, a total of 450, died in the hospital. In the first decennium, the number of males admitted was 812, of females, 751, the former considerably predominating. In the last decennium, the number of males was 1291, of females 1322, the latter predominating. The average number in the hospital has regularly increased from 107 in 1833, to 515 in 1852. Greatest number, at any one time, 552, in September, 1852.

3. *Bloomingdale Asylum.*—Dr. Nichols's report of the Bloomingdale Asylum, for 1851, is extremely brief, extending only to the length of *two pages.*

| | Men. | Women. | Total. |
|---|---|---|---|
| Patients remaining at the close of 1850 . | 50 | 60 | 110 |
| Admitted in 1851 . . . . . | 43 | 52 | 95 |
| Whole number . . . . . . | 93 | 112 | 205 |
| Discharged . . . . . . | 33 | 39 | 72 |
| Died . . . . . . . . | 6 | 5 | 11 |
| Remaining, Dec. 31 . . . . | 54 | 68 | 112 |
| Of those discharged, there were cured . | 17 | 26 | 43 |

*Causes of death.*—Epilepsy 3, serous apoplexy 2, "tuberculous disease, one chiefly affecting the bowels, the other the lungs," 2, puerperal peritonitis 1, arachnitis 1, "old cases of senile dementia, in one of which death appeared somewhat hastened by an attack of eczema, and the other by jaundice," 2.

Dr. Nichols resigned his office in the spring of 1852, and has since been appointed to the superintendence of the National Hospital for the Insane, about to be erected in the district of Columbia.

Dr. D. Tilden Brown, a gentleman well qualified for the place, was elected as successor to Dr. Nichols. In making up the report for 1852, although he has "followed in the footsteps of his predecessor," yet he hardly equals him in brevity, for the document extends to *three* pages.

| | Men. | Women. | Total. |
|---|---|---|---|
| Patients at the close of 1851 . . | 54 | 68 | 112 |
| Admitted in 1852 . . . . . . | 56 | 48 | 104 |
| Whole number in 1852 . . . | 110 | 116 | 226 |
| Discharged, died, and eloped . . | 58 | 49 | 107 |
| Remaining at the close of the year . | 52 | 67 | 119 |
| Of those discharged, there were cured . | 25 | 23 | 48 |
| Died . . . . . . . . | 11 | 7 | 18 |

*Causes of death.*—Paralysis 4, epilepsy 3, puerperal mania 2, exhaustion from prolonged excitement 2, apoplexy, inanition, extensive abscesses, dysentery, acute meningitis, 1 each; (2 not stated.)

"Seven patients died within ten days after admission, and five others after a stay varying from two to four weeks."

4. *Ohio Lunatic Asylum.*—Next upon our file to the report from Bloomingdale, lies its antipode in length, that of the Ohio Lunatic Asylum, for 1851. Here are ninety-five large and closely-printed pages, from the pen of Dr. S. Hanbury Smith, the extent of whose professional reading is probably as great as any other physician who has been connected with the institutions for the insane in this country.

| | Men. | Women. | Total. |
|---|---|---|---|
| Patients remaining Nov. 16, 1850 | 170 | 148 | 318 |
| Admitted in the course of the year | 133 | 150 | 283 |
| Whole number | 303 | 298 | 601 |
| Discharged | 153 | 147 | 300 |
| Remaining Nov. 16, 1851 | 150 | 151 | 301 |
| Of those discharged, there were cured | 77 | 86 | 163 |
| Died | 19 | 21 | 40 |

*Causes of death.*—Exhaustion of acute mania 5, chronic mania 6, phthisis pulmonalis 4, apoplexy 3, chronic peritonitis 3, dysentery 3, tabes mesenterica 3, inanition 3, chronic hepatitis 2, epilepsy, general paralysis, pneumonia, bilious colic, chronic colonitis, nephritis, gangrene of face, anasarca, 1 each.

"Five were brought in dying, and a sixth died on the road. Persons unaccustomed to see and treat such cases, are very apt to be deceived by the *apparent strength* of the patient, not suspecting that his unceasing exertions are rapidly wasting the oil of life."

"During the past year, no less than 23 persons were received, perfectly quiet and harmless, but in whom intellect was utterly extinct . . . . . . When, as has been so long and so advantageously practised in Continental Europe and Great Britain, the medical officers of State Lunatic Hospitals shall publicly lecture on medical disorders, and instruct a select class of advanced students; when a knowledge of the nature and treatment of insanity shall be looked upon as indispensable to the well-educated physician, and colleges shall make the possession of that knowledge a condition necessary to the obtaining of a diploma, then will establishments like this, mainly intended for *the cure of mental disorders, not the custody of the incurably insane*, cease to have their wards filled with hopeless cases, patients will neither be hurried off to die on the road, or immediately after their reception in the asylum, nor be kept at home until their cases have become hopeless."

We quote the following extracts from the remarks upon medical treatment.

"The asthenic character of disease, now becoming so marked in the great Western Valley, has been singularly prominent in the cases received last year. In no one was the idea of depletion entertained for an instant, by any of the medical officers of the institution; and those who had lost blood previously to their admission proved exceedingly difficult to restore, sank into hopeless dementia, or died. It must now be considered as a settled thing, that during the continuance of the present asthenic epidemic constitution, depletion is exceedingly hazardous, and commonly contraindicated in insanity; and in the very same forms of disease in which bloodletting was formerly so freely practised, the liberal use of stimulants is now required, tolerated, and proves eminently curative.

"In the treatment of acute cases of mania, the bowels have been first thoroughly cleared by appropriate medicine; such as a warm aloetic purgative, cathartic pills, or calomel and jalap, assisted by compound infusion of senna, and injections, as the case might be. Sometimes I employ an emeto-cathartic of one grain of tartar-emetic, ten grains of calomel, and twenty of ipecacuanha. After freely evacuating the bowels, they have been kept soluble by milder medicines. Immediately after the action of the cathartic, sometimes previously to, or during that action, the patient commences the use of from four to six ounces of wine, three or more times a-day, or an equivalent portion of brandy, together with abundance of the most nourishing and easily digested food. Excitement commonly ceases very quickly under the above treatment."

If the stimulus cease to have curative and produce pathogenitic effects, as indicated by a dry tongue, constipation, and cardiac irritability, it is stopped, and substituted by ferruginous tonics, of which Dr. Smith prefers the muriated tincture of iron.

"Only in some few cases, marked by extreme irritability, restlessness, and want of sleep, with moist, flabby, and tremulous tongue, natural or dilated pupil, frequent, irregular, and weak pulse, and cold relaxed skin, has opium been used to any extent; then commonly with wine, and always with excellent effect. In proportion as the general condition approaches that of a patient delirious from typhoid fever, have we found the combination of opium and tartar-emetic, as recommended by Graves, and in the form originally proposed by him, of service, though its use requires watching, to guard against ill-consequences from its depressing action."

"With regard to the treatment of puerperal mania, I have nothing new to offer. I shall content myself with expressing the opinion that bloodletting is eminently contraindicated. In this matter all authorities agree. Churchill says he 'has never found it advisable.' Prichard, that it 'is condemned by all practical writers, on whose judgment much reliance ought to be placed;' and Esquirol, Haslam, Gooch, and Burrows, are equally opposed to it. Even where local inflammation is discovered, there are other means of combating that besides bloodletting, which, if decided upon, must be used with the utmost caution; and, with the present epidemic constitution of disease, I should much fear even local depletion.

"Our main reliance is upon purgatives. If there is great excitement, I have much confidence in the already-mentioned combination of the potassio-tartrate of antimony, with opium, known as 'Graves's Mixture,' or muriate of ammonia, with digitalis; afterwards, if the head be not hot, full and repeated doses of opiates. Exhaustion is the great danger to be apprehended. This is to be combated with nourishing diet, easy of digestion, tonics, and stimulants, as wine, ammonia, turpentine. As a tonic and stimulant, I have a predilection for a combination of chinoidin and serpentaria, analogous to, but more active than Huxham's tincture; and in lesser degrees of debility, I employ valerian and arnica."

Dr. Smith states, that 37 of 108 married or widowed females admitted during the year, had become insane in consequence of childbearing; and 25 of the cases were *puerperal.* This "enormous" proportion serves as a text for remarks to a considerable length, in which he assumes that so great a prevalence of puerperal mania must act as a powerful genitive cause of insanity, by the transmission of an hereditary taint to the children.

Seven cases of insanity connected with disease of the ear, inflammation of the meatus-externus and the tympanum, were treated in the course of the year. Three of them were cured by curing this local disease. The fourth was a demented patient, in whom the otorrhœa produced maniacal excitement. The latter subsided in proportion as the former approximated restoration. The other three were under treatment, but two of them "have hitherto proved refractory."

Under the head of "Critical Phenomena," five cases are related, in which, during the course of the year, the patients were cured of their psychical disorder by diarrhœa; four, by "boils;" one, by "sloughing sores, especially on the lower limbs;" and two by "cachectic sores."

"A long experience," says Dr. Smith, "of the great value of the preparations of chlorine in adynamic conditions, caused by or accompanied with a presumably *septic* change in the blood, has led me to make trial of them in those forms of mental disease associated with an unusual lividity and coldness of the lips, hands, and sometimes the tip of the nose, evidently due to an embarrassed capillary circulation, and that, as I conceive, ascribable to some such morbid condition as that above mentioned. In such cases, the chlorate of potassa is the preparation I prefer, and the observations of the last year have fully confirmed the opinion of its value which I entertained. Again and again has its use corrected the condition of the circulation in question, when all other means had been tried in vain; speedily removing or diminishing the lividity, coldness, and sluggish movement, with a correspondent improvement in the health of body and mind. I commonly prescribe it in doses of two or three grains, quickly increasing to ten grains or more, dissolved in two or three ounces of camphor-water, two or three times a-day. Occasionally it may with advantage be administered in infusion of valerian, arnica, or with almost any other medicine indicated."

"In four cases, two of dementia and two of mania, caused by masturbation, the tincture of the muriate of iron, in large doses, was found to be the best anaphrodisiac we could employ, appearing to be the real agent in the cures which followed its use." The nymphomania of a case of recurrent mania was rapidly cured by bromide of potassa, in fifteen-grain doses, three times a-day.

Arranged under separate cautions in this elaborate Report, among them the "Impropriety of deceiving the insane," and "On the treatment of patients after they return home," the reader will find much interesting and useful matter. Since the publication of it, the hydra of politics has crawled, in its slimy pathway, into the domain of philanthropy, benevolence, and charity, in Ohio, and driven from their places all the officers, not of the lunatic asylum alone, but of all the state institutions in the city of Columbus. Ohio had previously taken and sustained an exalted position in regard to those of her citizens who, suffering under the defects and diseases to which our race are liable, demanded or merited that assistance which can best be afforded at public institutions. The whole expense of the lunatic asylum, original and current, and, if we mistake not, of some, if not all, of the other benevolent establishments, have been and are defrayed from the public treasury. So noble a precedent ought not to have been soiled, its merit dimmed, annulled, by one which, in the minds of all who rightly understand the nature of the duties of the officers of an institution for the insane, can but be considered as imprudent, unwise, unjust, ignoble, and seriously detrimental to the welfare of those for whose benefit those institutions are founded.

We are writing of *principles*, not of *men*. For aught we know, the places of the removed officers have been supplied by persons equally meritorious, although they lack the important element of experience. But if the principle be established that rotation in office, at public beneficent institutions, must correspond with the changes in national, State, or municipal politics; that the moneys devoted to charitable objects must be converted into "loaves and fishes" for the maws of political cormorants, then farewell to all progress in those institutions, to the hopes and aspirations of the philanthropist, and to the best interests of the insane, the blind, and the deaf and dumb. Apathy of officers, carelessness of police, looseness of administration, neglect of patients or other inmates, and general deterioration, must be the inevitable results. May Ohio soon retrace the path in which, by a humiliating descent, she has taken the initiative; and may all other States contemplate this precedent in its proper light, that they may shun and not follow it!

5. *The Illinois State Hospital for the Insane* is at Jacksonville, and under the superintendence of Dr. J. M. Higgins. The second biennial report was issued before the buildings were sufficiently complete to receive patients and consequently is principally devoted to financial affairs. From the third report, which closes with the 1st of December, 1852, we learn that the hospital was opened for the reception of patients on the 3rd of November, 1851.

| | Men. | Women. | Total. |
|---|---|---|---|
| Patients admitted | 73 | 65 | 138 |
| Discharged cured | 18 | 16 | 34 |
| Died | 1 | 5 | 6 |
| Remaining, Dec. 1, 1852 | 43 | 39 | 82 |

In view of the fact that about two hundred and forty applications for the admission of patients—enough to fill the present building—have already been made, and that the means of classification are imperfect, Dr. H. recommends the erection of two additional receding wings, as was contemplated in the original design of the establishment.

The barn was burned in July last, having been set on fire by one of the patients, intentionally, so far as appears. The buildings are to be warmed by steam, on a similar plan to that of the hospital at Trenton. The boilers are in a detached building, ninety feet distant from the principal edifice.

The amount of work which, according to the Report, has already been performed by the patients, is a sufficient evidence that a proper estimation of manual labour, as a hygienic and restorative means, is entertained by the superintending officer of the institution. (The above account of the American asylums is from the pen of Dr. Pliny Earle, and is extracted from the July number of the "American Journal of Medical Sciences," edited by Dr. Hays.)

---

